# Microextraction Techniques Coupled to Liquid Chromatography with Mass Spectrometry for the Determination of Organic Micropollutants in Environmental Water Samples

**DOI:** 10.3390/molecules190710320

**Published:** 2014-07-16

**Authors:** Mª Esther Torres Padrón, Cristina Afonso-Olivares, Zoraida Sosa-Ferrera, José Juan Santana-Rodríguez

**Affiliations:** Departamento de Química, Universidad de Las Palmas de Gran Canaria, 35017, Las Palmas de Gran Canaria, Spain; E-Mails: miriam.torres@ ulpgc.es (M.E.T.-P.); cristina.afonso102@alu.ulpgc.es (C.A.-O.); zoraida.sosa@ulpgc.es (Z.S.-F.)

**Keywords:** organic micropollutants, water samples, pesticides, pharmaceuticals, personal care products, microextraction techniques

## Abstract

Until recently, sample preparation was carried out using traditional techniques, such as liquid–liquid extraction (LLE), that use large volumes of organic solvents. Solid-phase extraction (SPE) uses much less solvent than LLE, although the volume can still be significant. These preparation methods are expensive, time-consuming and environmentally unfriendly. Recently, a great effort has been made to develop new analytical methodologies able to perform direct analyses using miniaturised equipment, thereby achieving high enrichment factors, minimising solvent consumption and reducing waste. These microextraction techniques improve the performance during sample preparation, particularly in complex water environmental samples, such as wastewaters, surface and ground waters, tap waters, sea and river waters. Liquid chromatography coupled to tandem mass spectrometry (LC/MS/MS) and time-of-flight mass spectrometric (TOF/MS) techniques can be used when analysing a broad range of organic micropollutants. Before separating and detecting these compounds in environmental samples, the target analytes must be extracted and pre-concentrated to make them detectable. In this work, we review the most recent applications of microextraction preparation techniques in different water environmental matrices to determine organic micropollutants: solid-phase microextraction SPME, in-tube solid-phase microextraction (IT-SPME), stir bar sorptive extraction (SBSE) and liquid-phase microextraction (LPME). Several groups of compounds are considered organic micropollutants because these are being released continuously into the environment. Many of these compounds are considered emerging contaminants. These analytes are generally compounds that are not covered by the existing regulations and are now detected more frequently in different environmental compartments. Pharmaceuticals, surfactants, personal care products and other chemicals are considered micropollutants. These compounds must be monitored because, although they are detected in low concentrations, they might be harmful toward ecosystems.

## 1. Introduction

The most representative chromatographic procedures for analysing micropollutants in water samples are based on multiresidue analysis with gas chromatography (GC). This instrumental technique requires volatile, thermally stable compounds, and many of the substances of interest in environmental samples tend to be adsorbed and decomposed on the columns or injector. Therefore, derivatisation reactions must be used [1].

Liquid chromatography (LC) and ultra-high-performance liquid chromatography (UHPLC) are now being used in combination with mass spectrometry (MS) for target analytes and for identifying nontarget analytes that are highly polar and non-volatile and have high molecular weights, making them incompatible with GC. Consequently, both the targeted and non-targeted analytes can be analysed or identified within a single analytical run. Therefore, liquid chromatography-mass spectrometry (LC-MS) combined with a sample pre-concentration/clean-up step is employed due to its excellent sensitivity and selectivity [2].

Sample treatment and enrichment processes are crucial during environmental analyses because the concentrations typically found in environmental waters are very low and the matrices are highly complex. Sample preparation may include clean-up and pre-concentration procedures to ensure that the analytes are found at a suitable concentration level.

Liquid-liquid extraction (LLE) and solid-phase extraction (SPE) are exhaustive traditional preparation techniques used to extract and pre-concentrate different families of analytes from environmental water samples. The need to reduce solvent volumes and to avoid using toxic organic solvents during LLE and SPE has led to adaptations of existing sample-preparation methods toward the development of new approaches. Consequently, miniaturisation has become a key factor while pursuing these objectives, and new techniques have been developed.

Microextraction techniques are generally defined as non-exhaustive sample preparation methods that utilise a very small volume of the extracting phase (in the range of µL) relative to the sample volume. Analytes are extracted using a small volume of a solid or semi-solid polymeric material through solid-phase microextraction (SPME) or of a liquid through solvent microextraction (SME). Despite the substantial structural differences between both techniques, they share similar features because they are both microextraction approaches [3]. Both methods are useful alternatives for sample preparation due to their simplicity, effectiveness, low cost, minimal solvent use and excellent abilities to clean up samples.

In this work, we review some of the most commonly used microextraction techniques and their applications toward the determination of some families of micropollutants in environmental liquid samples using mainly LC-MS.

Until the mid-1990s, the organic trace analysis of water mainly focused on persistent organic pollutants (POP), such as polychlorinated biphenyls (PCBs), polyciclic aromatic hydrocarbons (PAHs), organochlorine pesticides, *etc.*, based on their physicochemical characteristics (hydrophobicity, bioaccumulation and biomagnification through the trophic aquatic chain). Most of these substances have been banned, and their environmental concentrations are strictly controlled. However, interest in the fate and role of organic micropollutants, which they are present in the aqueous environment in nanograms or micrograms per litre, has increased. Many of these compounds are employed as household chemicals. Several pharmaceutical drugs, disinfection agents, pesticides and different personal care products can be included in this group [4], and these chemicals are also called emerging contaminants. This term refers to compounds that were not considered or known to be significant in terms of distribution and/or concentration in the past but are now widely detected [5]. Micropollutants include substances such as pharmaceuticals, drugs of abuse, biocidal compounds, food additives, cosmetic ingredients or detergents [6]. These compounds are often released from various municipal, agricultural and industrial sources and pathways and have been detected in wastewater treatment plant (WWTP) effluents [7,8,9,10]. Moreover, increasing evidence suggests that many organic micropollutants are endocrine disruptor compounds (EDCs) found in various products, including plastic bottles, detergents, flame-retardants, food, toys, cosmetics, pesticides, *etc.* These organic micropollutants and their degradation products may be toxic and persistent and, despite being detected in low concentrations, could produce potentially harmful effects on ecosystems and human health [11,12].

The US Environmental Protection Agency (USEPA) published the final Contaminant Candidate List (CCL-3) in September 2009, which is a drinking-water priority-contaminant list used for regulatory decision-making and information collection. The contaminants listed are either known or anticipated to exist in drinking-water systems and will be considered for regulation. This final CCL-3 contains 104 chemicals and 12 microbial contaminants, including pesticides, disinfection by-products, chemicals used in commerce, waterborne pathogens, pharmaceuticals and biological toxins [13].

Similarly, the Water Framework Directive (WFD) sets the European Union (EU) strategy against the pollution of water by dangerous substances. The WFD provisions will require the Member and Associated States to establish programs to monitor water quality, review the effect of human activity on pollutants and perform an economic analysis of water use. In this context, an initial list of priority substances was published in 2001. This list was revised in 2008, coinciding with Directive 2008/105/EC; the latter document was related to the environmental quality standards in the field of water policy. A new list was published in 2011 [14]. In the future, some of these organic micropollutants might be candidates for introduction into the WFD list of priority substances.

## 2. Solid-Phase Microextraction

Arthur and Pawliszyn [15] introduced solid-phase microextraction (SPME), generating interest in microextraction techniques for analytical chemistry. When using SPME, the analytes are isolated based on the equilibrium between the sample matrix and the extractive coating after selecting an appropriate extractive phase and reducing the volume to remove as many of the unwanted compounds as possible. This strategy leads to efficient clean-up and minimizes the matrix effect during mass spectrometry detection, which is a serious concern in liquid LC–MS systems.

**Figure 1:**
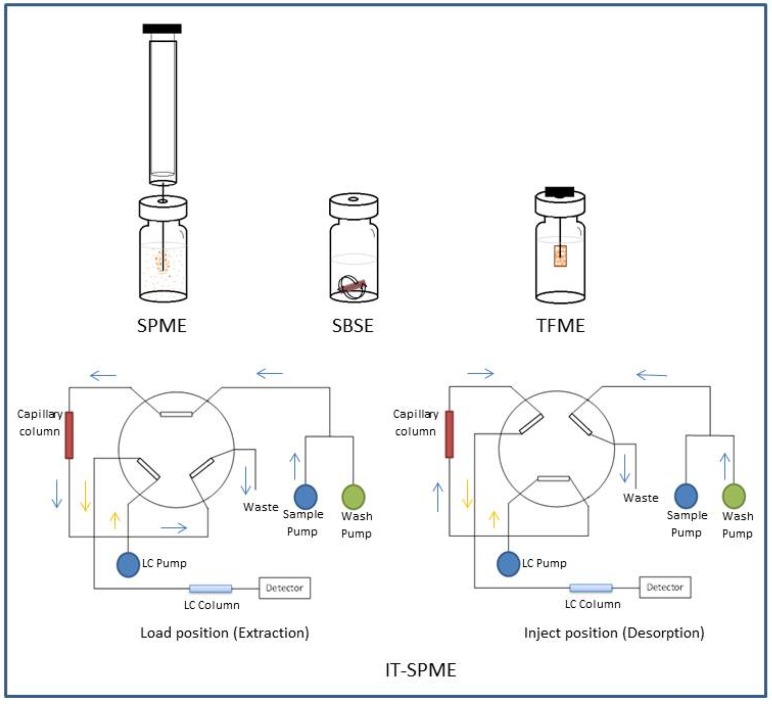
Scheme of some solid phase microextraction techniques.

SPME configurations can be classified into static and dynamic techniques. Static procedures are typically carried out in stirred samples, including fibre SPME, thin-film microextraction (TFME), rotating disk sorptive extraction (RDSE), stir bar sorptive extraction (SBSE) and dispersive SPME. Fibre SPME, which is the most common format for this technique, utilises a sorbent coating on the outer surface of a fused silica fibre to extract the analyte(s) from the sample matrix; this process occurs through direct immersion (DI-SPME) or from the sample headspace in a closed container (HS-SPME). The dynamic techniques include capillary microextraction (CME) techniques, such as in-tube SPME (IT-SPME), in-needle and in-tip microextraction configurations. SPME focuses mainly on the development of new coatings and novel analytical strategies that improve the sensitivity [16]. A schematic diagram of some configurations is shown in Figure 1.

Sol-gel technology was applied to prepare SPME fibres in 1997 [17]; since then, it has become one of the most popular approaches for preparing novel SPME coatings. This technology has already helped synthesise many novel sorbents for SPME with large surface areas, unique selectivity and high thermal and solvent stabilities; these characteristics contribute to the high sample pre-concentration factors. The versatility of these materials enables the creation of surface-bonded sorbent coatings on unbreakable fibre materials and on substrates with different geometrical formats. Sol-gel coatings are applied during the extraction of various analytes from different sample matrices in the fibre-SPME and in the SBSE configuration [18]. Therefore, a novel polar sol-gel precursor, cyanopropyltriethoxysilane (CNPrTEOS), was combined with PDMS for the SBSE of two non-steroidal anti-inflammatory drugs (NSAIDs) from aqueous samples [19].

Carbon nanotubes (CNTs) are interesting targets when studying new materials in SPME. CNTs are allotropic forms of graphitic carbon comprising a single rolled graphite lamella that forms a tube (single-wall carbon nanotubes, SWCNTs) or several single tubes arranged around a common axis (multi-wall carbon nanotubes, MWCNTs); the surface-to-volume ratios of these materials are significant [20,21]. A sol-gel amino-modified multi-walled carbon nanotube-PDMS (AMMWCNT-PDMS) was synthesised for use as a novel coating for the SBSE of phenols from environmental waters [22]. Metal-organic frameworks (MOF) are a new class of porous solid materials that are self-assembled by metal ions and organic ligands. Recently, Hu *et al.* [23] proposed a sol-gel coating for SBSE based on PDMS and a MOF to analyse oestrogens in environmental water samples.

The selectivity required for SPME can be provided through molecular imprinting (MIPs), as demonstrated by Koster *et al.* [24]. MIPs are polymeric materials with a high binding capacity and good selectivity against a target molecule purposely introduced during the synthetic process. MIPs are typically synthesised through the co-polymerisation of functional monomers and templates. The functional monomers should possess specific functional groups, and the templates are always the target analytes or their analogous compounds. Cross-linkers are also required to form rigid polymer networks that stabilise the cavities for the target molecules, making the polymer mechanically and thermally robust. Porogens are sometimes required to attain a porous morphology and thereby enhancing mass transfer [25].

A new polymerisation strategy called molecular imprinting solid-phase microextraction (MISPME) has been developed in different formats, such as MIP-coated fibres (polymeric membranes) and MIP rod-like fibres (polymeric monoliths). MISPME is a successful and novel microextraction technique that enriches the selected analytes from various real samples, including environmental samples [26]. Bisphenol A [27], phthalates [28] and triazines [29] in liquid samples have been detected through this strategy.

Ionic liquids/polymeric ionic liquids (ILs/PILs) are promising sorbent-coating materials designed to exhibit high selectivity for targeted analytes. ILs are salts with organic cations and organic/inorganic anions with melting points at or below 100 °C. These materials possess high thermal stability, tuneable viscosity and solvation capabilities and negligible vapour pressures. The primary advantage of using ILs as SPME sorbent coatings involves the ability to incorporate various substituents into the IL structure [30]. PILs are polymers synthesised from IL monomers that exhibit some advantages over ILs when used as coatings for SPME. PILs often possess higher viscosities and greater mechanical strengths compared to ILs but exhibit similar extraction selectivities [30]. Although studies of IL/PILs-based sorbent coatings in SPME have become extremely popular, the stability must be improved to enhance the robustness of the coating when studying new sorbent-loading methodologies and fibre surface modifications. Both ILs and PILs have been widely used as SPME coatings in numerous applications, especially for the analysis of water samples, through both direct immersion and headspace; all of these methods have been coupled to GC [30].

In-tube SPME, which is the capillary format of SPME, utilises a tubular extraction device that contains an extraction phase as a surface coating or monolithic sorbent bed. In-tube SPME is also known as capillary microextraction (CME) [18]. In this case, the sorbent medium plays the most significant role during sample preparation; it is highly selective for the target analyte and should be thermally and chemically stable, providing highly efficient extraction. Unlike SPME fibres, the coated capillaries are not commercially available. Toward that purpose, a small selection of commercially available GC columns is used [31]. Aufartová *et al.* [32] optimised this microextraction technique to extract oestrogens from environmental liquid samples using Carboxen and Supel-Q capillary columns. However, the low sorbent loading, which resulted from the thin stationary phase coatings in the used GC column segments, results in low sample capacity, impeding the pre-concentration step. In the last decade, sol-gel coatings and monolithic beds have been developed to solve the in-tube SPME problems (e.g., low sorbent loading) in order to overcome this format-related deficiency [18]. Micellar media have been used as alternatives to organic solvent during IT-SPME [33].

Similarly to SPME, stir bar sorptive extraction (SBSE) is also an equilibrium-based non-exhaustive sample-preparation technique. However, the major difference between SPME and SBSE is the high sorbent loading on the stir bars, which imparts increased sample pre-concentration capabilities. During stir bar sorptive extraction (SBSE), a magnetic stir bar coated with polydimethylsiloxane (PDMS), which has a larger surface area than a SPME fibre, is spun into an aqueous sample (or extract) for a selected long extraction time. Once the extraction step is completed, the stir bar is removed, a step that is usually performed manually, and a fraction of the concentrated extract is transferred to a GC system or diluted for LC analysis [34].

The feasibility of SBSE for pre-concentrating analytes with medium to low polarity and divergent volatility from essentially aqueous samples (or extracts) has been demonstrated [35,36,37], and the several advantages of SBSE compared to SPME in most of these applications have been described. However, this technique has not been as widely accepted as SPME due to the limited number of commercially available coatings and the difficulty of achieving full automation. Currently, efforts in this field are focused on the development of dual phase/hybrid twisters, where the conventional PDMS phase is combined with another sorbent to increase the selectivity and/or efficiency of the extraction process [19], or alternative new coating materials with improved analytical features, promoting the retention of polar compounds from complex matrices. An extensive review published by Gilart *et al.* [38] covers the state of novel commercial and in-house coatings for SBSE in recent years, particularly their application for the extraction of polar micropollutants from complex matrices.

Bar adsorptive micro-extraction (BAµE) is a novel static microextraction technique for trace analysis of polar compounds in aqueous media, which uses nanostructured materials (e.g., activated carbons or polymers), for each particular type of target compounds [39]. This new analytical approach, operates under the floating sampling technology and it has shown high effectiveness in many applications [40,41,42]. The major trends in SPME are moving toward the introduction of new selective coatings and devices to enhance the extraction efficiencies from complex matrices.

## 3. Solvent Microextraction

Solvent microextraction (SME) is a technique for sample preparation involving the extraction and concentration of liquid, gaseous and solid samples with solvent volumes in the µL or sub-µL range, thereby enabling high enrichment factors. The term liquid-phase microextraction (LPME) is also frequently used to describe this process [43]. This rapid inexpensive preparation technique uses minimal solvent volumes with negligible exposure to toxic organic solvents. LPME is normally performed using a small volume of a water-immiscible solvent and an aqueous phase containing the analytes of interest. From the introduction of the first paper on SME in 1996 [44] until now, different approaches have been developed in two broad categories: exposed solvent and membrane-protected solvent [45].

**Figure 2:**
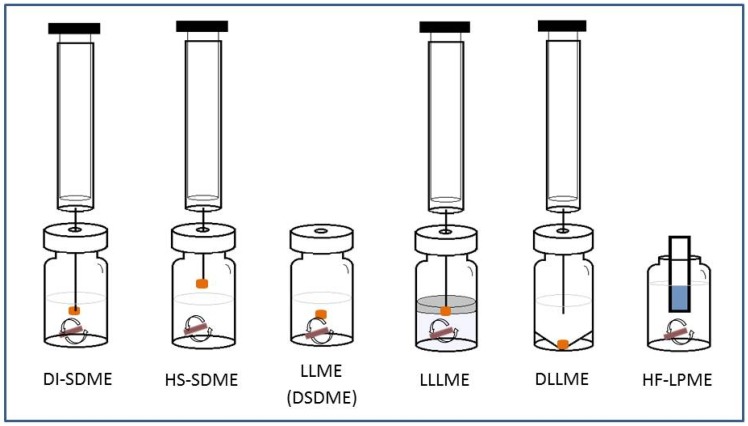
Scheme of some solvent microextraction techniques.

Exposed solvent techniques include single-drop microextraction (SDME), headspace single drop microextraction (HS-SDME), liquid-liquid microextraction (LLME, which is also called directly suspended droplet microextraction, DSDME), liquid-liquid-liquid microextraction (LLLME) and dispersive liquid-liquid microextraction (DLLME) [43,45] as shown as in Figure 2.

Single Drop Microextraction (SDME) is a miniature liquid-liquid extraction: a drop of water immiscible organic extracting solvent (approximately 1–10 µL) is suspended from a syringe into the liquid or gaseous sample medium. After extraction, the liquid extractant is drawn back into the microsyringe and used directly to determine the analytes via GC. SDME is not exhaustive, and only a small fraction of the analyte is extracted and pre-concentrated for analysis [43].

Headspace (HS-SDME) enables the extraction and pre-concentration of volatile or semi-volatile compounds into a microdrop exposed to the headspace above the sample. The drop remains at the tip of the microsyringe throughout the extraction period before being retracted back into the microsyringe. In this mode, the analytes are distributed between three phases: the water sample, headspace and organic drop. HS-SDME can achieve a high degree of extract clean-up because non-volatile compounds and high-molecular-weight species are not extracted [43,45]. In all cases, GC is used to determine the target analytes.

The major disadvantages of both techniques are the susceptibility of the drop toward dislodging during sampling, the size limitations of the drop and the volatility of the extraction solvent [45]. To resolve these drawbacks, air is deliberately introduced with the solvent drop, leading to a larger solvent surface area. The bubble also tends to support high-density solvents (e.g., CHCl_3_), which tend to dislodge due to their weight [46]; otherwise, water-insoluble ionic liquids (ILs) are used. The latter are alternatives to organic solvents due to their high viscosity and surface tension, which helps the formation of a stable drop with a much larger volume [47]. However, the instability of the ionic liquid drop at the end of the needle remains the most significant limitation of SDME when coupling this technique to high-performance liquid chromatography (HPLC). Small solvent volumes (microliter level) are not sufficient for performing highly sensitive liquid chromatography determinations.

Another alternative to organic solvents are the supramolecular assembly-based coacervates (e.g., surfactant micelles) that have been applied during analytical techniques to extract various organic compounds before their separation by LC. A vesicular-based coacervate was prepared by mixing decanoic acid in tetrabutyl ammonium hydroxide and distilled water and was used as the solvent in SDME. This technique, which is called single-drop coacervative microextraction (SDCME) [48], has been applied to extract chlorophenols from wastewater, superficial water from a reservoir and groundwater before liquid chromatography determination.

During liquid-liquid microextraction (LLME), which is also called directly suspended droplet microextraction (DSDME), 10–100 µL of an organic solvent is added to the centre of the stirring vortex of an aqueous sample. The direct interface between the solvent and water rapidly extracts and concentrates the analytes in the organic solvent, which is subsequently removed with a capillary tube or syringe and injected into a chromatographic system for analysis.

Liquid-liquid-liquid microextraction (LLLME) is similar to LLME. This two-step process first extracts an ionisable solute into an organic layer before extraction and trapping it in a second aqueous layer with a pH capable of ionising the solute. Typically, this technique is used to extract acidic, basic or polar analytes from water into an acidic (for basic analytes) or basic (for acidic analytes) acceptor solution [45]. For example, Lin *et al.* [49] utilised LLLME to extract long-chain alkylphenols, such as 4-*t*-butylphenol, 4-*t*-octylphenol, 4-*n*-nonylphenol and bisphenol-A, from water.

Dispersive liquid-liquid microextraction (DLLME) is a simple and rapid microextraction method that uses µL volumes of a dense organic solvent with a few mL of dispersive solvent. A cloudy solution is formed when the appropriate mixture of extraction and dispersive solvents is injected into an aqueous sample containing the analytes of interest. After centrifuging the cloudy solution, the phase at the bottom of a conical tube is recovered and analysed by GC [43,45]. This microextraction technique is useful for non-polar analytes; it generally requires a halogenated solvent (tetrachloroethylene or carbon tetrachloride) and a water-soluble co-solvent (methanol (MeOH), acetone or acetonitrile (ACN)) that increases the solubility of the extraction solvent in water.

Solvents with a lower density than water (octanol, toluene) have also been applied in DLLME [50,51,52]. Too, a mixture of two water-immiscible solvents (polar and non-polar) and auxiliary solvent was used for the extraction of ion-pair complexes from water samples in order to ensure that the resulting mixture has a density higher than that of water, [50].

In recent years, interest in DLLME has focused on using low-toxicity solvents with convenient and practical procedures. In this context, Sun *et al.* developed a new mode of IL-DLLME based on hydrophilic and hydrophobic ionic liquids to determine two acidic phenolic compounds (2-naphthol and 4-nitrophenol) in environmental water [53].

Membrane-protected solvent techniques include hollow-fibre-protected 2-phase microextraction [HF(2)ME], which is often called LPME or hollow-fibre LPME in the literature [45]; during this procedure, a porous polypropylene hollow fibre contains the extraction solvent within its pores and lumen. The organic solvent forms a thin layer within the wall of the hollow fibre, and the fibre is then inserted into a sample vial filled with the aqueous sample of interest. The analytes are extracted from the aqueous sample through the organic phase into the pores of the hollow-fibre before entering the acceptor solution inside the lumen [43].

Hollow-fibre-protected phase microextraction mode is HF(3)ME, often referred to as again LPME or 3-phase LPME. In this case, a water-insoluble, non-polar solvent saturates the wall of the fibre, whereas the lumen contains an acid or base; this system irreversibly extracts the analytes [45]. Gure *et al.* proposed a three-phase hollow-fibre liquid-phase microextraction combined with a LC method using diode array detection (DAD) to determine six sulfonylurea herbicides, specifically triasulfuron, metsulfuron-methyl, chlorsulfuron, flazasulfuron, chlorimuron-ethyl, and primisulfuron-methyl, in environmental water samples [54].

Similar to other microextraction techniques, hollow fibres can be modified. A technique employing ionic liquids during HF-LPME is hollow fibre membrane liquid–liquid–liquid microextraction (HFM-LLLME) [55]. For another derivative of HF-LPME, the membrane pores were filled with a solvent containing dispersed multiwalled carbon nanotubes (MWCNT). This technique is called hollow fibre solid–liquid phase microextraction (HF-SLPME) and demonstrates good extraction efficiency with organic analytes extracted from aqueous samples [47].

## 4. Applications of Microextraction Techniques to the Determination of Organic Micropollutants

GC is one of the most important techniques used during environmental analyses. However, in recent years, LC-MS have become popular for identifying unknown contaminants or improving the selectivity for known analytes [2]. 

In this section, we describe some reported methods for determining organic micropollutants that couple the described microextraction techniques with LC-MS and UHPLC-MS. We selected some micropollutants families based on their interest and presence in environmental liquid samples. Table 1, Table 2 and Table 3 summarise the main characteristics of the selected microextraction techniques (matrix, time, some analytical parameters…).

### 4.1. Pesticides

Scientific advances have created the hundreds of synthetic organic compounds used as pesticides. Their physicochemical properties and widespread use in agriculture, antifouling and household products explain their pervasiveness in aquatic environments, including wastewater [56], surface and ground water [57] and seawater from coastal areas [58].

In the field of environmental water policy, annex I of Directive 2008/105/CE includes the maximum allowable concentration of some priority substances. The maximal average annual concentrations authorised for surface water can vary from ng·L^−1^ to μg·L^−1^.

In this case, microextraction techniques are often used to analyse pesticides, as shown in Table 1.

Solid-phase microextraction (SPME) has been used to extract pesticides. For example, Ugarte *et al.* [59] used this technique with a commercial polydimethylsiloxane/divinylbenzene (PDMS/DVB) fibre coupled to a high-performance liquid chromatography (HPLC) system with subsequent detection by inductively coupled plasma-mass spectrometry (ICP-MS) to determine different organotin compounds in fresh- and seawater samples from leisure ports. The optimal method provided limits of detection (LOD) between 6 and 185 ng·L^−1^.

A high-throughput method based on thin-film solid-phase microextraction (TF-SPME) and liquid chromatography mass spectrometry was developed by Boyaci *et al.* [60] to simultaneously quantify nine benzylic and aliphatic quaternary ammonium compounds in aqueous samples; these compounds are used as disinfectants. TF-SPME has a coating with a higher surface area/volume ratio and is particularly useful when analytes are found in trace amounts, in complex matrices, or in the presence of a binding matrix with low concentrations of free analytes available for extraction. This method was validated according to the Food and Drug Administration (FDA) criteria using river water. The accuracy achieved was near 100%, and the limits of quantification (LOQs) defined by the lowest calibration points ranged from 0.01 to 0.50 µg·L^−1^.

In-tube solid phase microextraction (IT-SPME) is the most commonly used microextraction technique for assessing organic micropollutants. Wu *et al.* [61] used a custom polypyrrole (PPY)-coated capillary to assay polar pesticides (six phenylurea and six carbamates pesticides) in spiked water. The extraction conditions were optimised, particularly the stationary phases; the custom-made capillaries and several commercial capillaries were compared. The LODs of this method for the studied compounds ranged from 0.01 to 1.2 µg·L^−1^.

Masiá *et al.* [62] proposed a multiresidue analytical method for the pesticides included in the Water Frame Directive 2000/60/EC (WFD) that combines IT-SPME with a GC TRB-5 capillary column and ultra-high-performance liquid chromatography tandem mass spectrometry (UHPLC–MS/MS). This method exhibited good linearity over the assayed range and LODs between 0.025 and 2.5 µg·L^−1^. This method was applied to several water samples from different sources, demonstrating the on-line enrichment of the analytes with minimal sample manipulations; these compounds were identified, and their concentrations were quantified as low levels in units of parts-per-billion.

Stir bar solid extraction (SBSE) was used by Giordano *et al.* [63] to extract 16 pesticides from surface water samples. This method was validated in spiked surface water samples, and the obtained LODs ranged from 0.01 to 1.0 μg·L^−1^.

Finally, Pedrouzo *et al.* [64] optimised an UHPLC-MS/MS method using SBSE to analyse the antimicrobial compounds triclosan and triclocarban in surface and wastewaters. The LODs of the analytical method were 2.5 ng·L^−1^ for river water and 5–10 ng·L^−1^ for the effluent and influent sewage waters. Triclosan was found at levels <LOQ in river waters and was commonly below 25 ng·L^−1^ in the sewage effluent.

Another technique for determining pesticides is dispersive liquid-liquid microextraction (DLLME). Caldas *et al.* [65] applied this microextraction technique using acetonitrile as the dispersive solvent and carbon tetrachloride as the extraction solvent to extract and pre-concentrate different classes of pesticides (carbofuran, clomazone and tebuconazole) in aqueous samples. Under the optimal conditions, the recoveries of the pesticides in the spiked water ranged from 62.7% to 120.0%; the LOQs of the method were 0.02 µg·L^−1^ after accounting for the 50-fold pre-concentration.

Zheng *et al.* [66] developed a novel dispersive liquid–liquid microextraction based on the solidification of a floating organic droplet (DLLME-SFO) to analyse triclosan and its degradation product in real water samples using acetonitrile as the dispersive solvent and 1-dodecanol as the extractant. The major difference between DLLME-SFO and DLLME is that the extractant used in the former has a low melting point and hypotoxicity. The extraction solvent used in this work has a low density, low volatility, low toxicity and proper melting point near room temperature. The extractant droplets can be collected by solidification at a lower temperature. The LODs of this microextraction technique in combination with LC–MS/MS ranged from 0.002 to 0.02 µg·L^−1^. Wide linearities, good precisions and satisfactory relative recoveries (83%–119%) were obtained.

Dispersive liquid-phase microextraction (DLPME) has also been used to extract these compounds. A recent study by Zhou *et al.* [67] described a temperature-controlled ionic liquid dispersive liquid-phase microextraction (IL-DLPME) developed to enrich and to determine triazine herbicides in water samples using 1-octyl-3-methylimidazolium hexafluorophosphate ([C_8_MIM][PF_6_]) as the extractant. Under the optimal conditions, the LODs ranged from 0.05 to 0.06 μg·L^−1^. Different real water samples were analysed, and the experimental results showed that the spiked recoveries were satisfactory. This same technique was used previously by Zhao *et al.* [68] to analyse triclosan and triclocarban in environmental water samples. Methanol was used as the dispersant, and [C_6_MIM][PF_6_] was the extractant. This method was used to analyse real environmental water samples with satisfactory results. The average recoveries of the spiked compounds ranged from 70.0% to 103.5% with LODs from 0.040 to 0.58 μg·L^−1^.

### 4.2. UV Filters Including Benzotriazoles

Organic ultraviolet (UV) filters have been employed for decades during the formulation of personal-care products (PCPs). Although they were initially designed for sunscreen formulations, they are added to other daily cosmetic products to prevent the harmful effects of UV exposure [69].

Benzotriazole UV stabilisers (BUVSs) are one of the most commonly employed types of UV filters. These derivatives of benzotriazole absorb the full spectrum of UV light including UV-A (320–400 nm) and UV-B (280–320 nm); they are used in many PCPs, as well as for several other purposes, such as corrosion inhibitors in dishwasher detergents and UV-light stabilisers in plastics or dental restorative materials [69].

Some of these compounds have been identified in List 3 by the United States Environmental Protection Agency (USEPA) as ingredients of unknown toxicity [70].

Stir bar solid extraction (SBSE) is the most common microextraction technique, as shown in Table 2. Pedrouzo *et al.* [64] developed a SBSE method with liquid desorption and UHPLC–(ESI)MS–MS to extract and analyse four UV filters (2,2-dihydroxy-4-methoxybenzophenone, benzophenone-3, octocrylene, and octyldimethyl-*p*-aminobenzoic acid). The method was sensitive enough to determine these compounds at trace levels in environmental waters. In river waters, benzophenone-3 ranged from 6 to 28 ng·L^−1^. Benzophenone-3 ranged from 75 to 127 ng·L^−1^ in the influent sewage and fell below 25 ng·L^−1^ in the effluent sewage.

Montesdeoca-Esponda *et al.* [71] optimised a SBSE-based method using polydimethylsiloxane (PDMS Twister^®^) with liquid desorption to extract benzotriazole UV stabilisers from water samples for analysis by ultra-high performance LC with MS/MS detection. The optimised method was applied to seawater and wastewater samples with good selectivity, high sensitivity and limits of quantification ranging from 61.5 to 184 ng·L^−1^. Recoveries between 68.4% and 92.2% were achieved for the more polar compounds.

Recently, Gilart *et al.* [72] optimised SBSE methods coupled with liquid chromatography tandem mass spectrometry (LC–MS/MS) using two new commercially available polar coatings consisting of polyacrylate (PA) with polyethyleneglycol (PEG) (Acrylate Twister^®^) or PEG-modified silicone (EG Silicone Twister^®^); these materials were compared to the classic coating based on polydimethylsiloxane (PDMS Twister^®^) for the extraction of a group of pharmaceuticals and personal care products (PPCPs), including UV filters, from wastewater samples. The EG Silicone coating extracted some of the polar compounds more efficiently while improving the sorption of nonpolar compounds compared to the other two coatings.

### 4.3. Alkylphenols and Bisphenol A

Alkylphenols (APs), their ethoxylated derivatives (APEOs) and bisphenol A (BPA) are endocrine disrupting compounds (EDCs) because these compounds can alter the endocrine system of living organisms, including humans. APs are used as surfactants, whereas BPA is a monomer used during the manufacture of plastics. The main sources of these compounds in the aquatic environment are WWTPs where domestic and industrial wastewaters converge. In general, the concentrations of these substances in liquid environmental samples range from a few ng·L^−1^ in relatively clean samples, such as surface water, to hundreds of ng·L^−1^ in more complex samples, such as WWTP samples.

During the last decade, the number of publications using liquid chromatography coupled to mass spectrometry for detection has grown significantly; the most common extraction technique is solid-phase extraction [73]. However, some microextraction techniques have been optimised for these target compounds, as shown in Table 2.

Salgueiro *et al.* developed and validated a method that determines APs and BPA in seawater simultaneously [74]. This procedure was based on dispersive liquid–liquid microextraction (DLLME): 1-octanol and a small volume of the seawater sample were combined with liquid chromatography–electrospray ionisation tandem mass spectrometry in the negative mode (LC–ESI-MS/MS). The recoveries were satisfactory (approximately 84%–104% for all compounds). The LOQs ranged between 0.005 and 0.03 µg·L^−1^; therefore, the levels established in Directive 2008/105/EC [14] were achieved.

Recently, Fabregat *et al.* [75] developed a new method to extract and quantify APs in complex matrix water samples rapidly using HF-LPME with ultra-high liquid chromatography tandem mass spectrometry (UHPLC-MS/MS). In this case, 1-octanol was used as the acceptor phase, and an enrichment factor of 800 was obtained. The quantification was carried out through isotope pattern deconvolution, which allowed the quantification of the concentrations of both compounds without a calibration graph, thereby decreasing the total analysis time. Combining HF-LPME and UHPLC-MS/MS enabled the validation of this methodology at the legislated levels, achieving LOQs of 0.1 µg·L^−1^ and recoveries from 97% to 109%.

### 4.4. Perfluorinated Compounds

Perfluorinated compounds (PFCs) comprise a class of artificial, fully fluorinated organic compounds and may exhibit both hydro- and lipophobic characteristics. These anthropogenic compounds have numerous applications as surfactants, fire-fighting foams, textiles, *etc.*; their entry into the medium may be attributed to industrial discharge, the degradation of precursor compounds and the use of articles containing them. These bioaccumulative substances are abundant in the aquatic environment, where they might adversely affect humans and animals [76]. Although these compounds were first produced in the 1950s, the wide distribution of PFCs in the environment was not apparent until 2000. Among these compounds, perfluorooctane sulfonate (PFOS) and perfluorooctanoate (PFOA) have received the most attention in recent years. PFCs are detected in both waste- and surface waters at ng·L^−1^–µg·L^−1^ levels [77], as well as in open ocean waters [78].

PFCs are determined using LC-MS [79] after extraction and pre-concentration through methods such as SPE. Few studies have used the microextraction techniques described in this review, as shown in Table 2. 

In this context, three different microextraction methods have been developed. IT-SPME [80] and SPME [81] were used to analyse PFOS and PFOA. The results were very similar in both cases because these techniques have the same theoretical basis. SPME fibres were prepared by the chemical bonding of a sol-gel precursor to anodised Ti, whereas IT-SPME utilised a CP-Pora PLOT amine capillary column. It is possible that IT-SPME (RSD below 3.7%) was slightly more accurate than SPME (RSD below 5.2%), but the recoveries with both methods were above 81%, and the LODs were approximately a few ng·L^−1^ in both cases.

LLME with a mild emulsification procedure, specifically vortex mixing, was used by Papadopoulou *et al.* [82] to determine the PFOS concentration in aqueous environmental matrices with an octanol-like acceptor phase. The recoveries under the optimal conditions ranged from 90.8% to 105.1%, and the LOD was satisfactory (1.6 ng·L^−1^).

### 4.5. Hormones

Residual hormones have become a source of major concern because they can affect the biological activity of non-targeted organisms. These compounds are a potential risk for wildlife and humans through the consumption of contaminated food or water. The most potent active EDCs present in the environment are steroids, which can be formed naturally by humans and wildlife or produced synthetically. At low concentrations, steroidal hormones alter the endocrine system, changing the growth, development, and/or reproduction of exposed animals. These changes may be expressed later in the life cycle or in future generations. Therefore, determining the fate and distribution of steroids and their conjugates in the environment is critical because they are potential sources of active oestrogens after dissociation in wastewater treatment plants or the influx of treated wastewater directly into surface waters [83].

Analytical methods with high sensitivity, selectivity and resolution must be developed to determine low concentrations of these substances and to overcome matrix complexity.

Although there are many extraction techniques for liquid samples, solid-phase extraction is the most common. Other miniaturisation techniques have also been employed to analyse hormones in water samples: SPME, IT-SPME, DLLME and SBSE [84]. However, LC coupled with DAD or a fluorescence detector (FD) was used for detection; a unique study employing microextraction techniques coupled to LC-MS was published by Mitani *et al.* [85] (Table 3). Five oestrogens were analysed in environmental waters by IT-SPME with a Supel-Q PLOT capillary column. The recoveries under the optimal conditions ranged from 86.1% to 106.8%, and the LOD ranged from 2.7 to 11.7 ng·L^−1^.

### 4.6. Pharmaceuticals

The presence of pharmaceutical compounds in aquatic media is a challenge during environmental monitoring. These substances are pervasive in rivers, lakes and oceans due to their dispersion through wastewater [86]. Although many countries use advanced technologies, such as ozonation, reverse osmosis, and granular active carbon, to treat potable water, some compounds resist treatment [87].

Despite the existence of numerous pharmaceutical compounds, few studies utilised liquid chromatography with mass spectrometric detection (LC-MS) with microextraction techniques, as shown in Table 3.

Two studies have used SPME to extract similar antibiotics compounds in liquid samples. Balakrishnan *et al.* [88] used Carbowax/divinylbenzene (CW/DVB) fibres to extract ten sulphonamide antibiotics from different wastewater samples, revealing a viable method for overcoming the matrix effects. McClure *et al.* [89] optimised a SPME method using Carbowax-template resin (CW/TPR) fibres to collect antibiotics (five of nine compounds were sulphonamides) in influent and effluent samples simultaneously. The LODs obtained in this study were better (ng·L^−1^) than those obtained by Balakrishnan *et al.* [88].

A multi-residue analysis of the pharmaceutical compounds in wastewater through dual solid-phase microextraction (dSPME) was realised by Unceta *et al.* [90]. Two CW/TPR fibres with different pH values were used to obtain excellent recoveries (89.2%–109.7%) for numerous compounds.

Strittmatter *et al.* [91] developed an analytical method by combining C_18_/SCX mixed thin-film microextraction (TFME) and desorption electrospray ionisation mass spectrometry (DESI-MS) to determine pharmaceuticals in aqueous samples. Combining both techniques improves the analysis time considerably compared to traditional liquid chromatography mass spectrometry (LC-MS). The results were compared, and good agreement was found through a concentration range spanning three orders of magnitude. Serious matrix effects were observed in treated wastewater, but the lower limits of detection were still in the low ng·L^−1^ range.

IT-SPME was used by Mitani *et al.* [92] to extract five fluoroquinolones (FQs) from environmental waters using a fully automated method with a Carboxen 1010 PLOT capillary column-like IT-SPME system coupled to a liquid chromatography-tandem mass spectrometry (LC-MS/MS) system. The extracted compounds were easily desorbed using the mobile phase. The LODs of the five FQs ranged from 7 to 29 ng·L^−1^. The IT-SPME method had between 60-94-fold higher sensitivity than the direct injection method.

Ohcho *et al.* developed an IT-SPME method using a Carboxen 1006 PLOT capillary column to simultaneously determine 15 non-steroidal anti-inflammatory drugs (NSAIDs) in environmental water [93]. The LODs of the NSAIDs ranged from 5 to 65 ng·L^−1^. This method could be used to analyse surface and wastewater samples without any pre-treatment or interference peaks. Although IT-SPME has achieved good recoveries (above 80%) and limits of detection (ng·L^−1^) for the analysed compounds, it is rarely used with LC-MS detection.

In recent years, stir bar solid extraction (SBSE) has been used to determine different pharmaceutical compounds. SBSE with different commercial external coatings, such as PDMS (polydimethylsiloxane), EG Silicone (ethylene glycol-silicone) and PA (acrylate), can be used [72]. Moreover, new SBSE coatings have been prepared.

In this context, Bratkowska *et al.* [94] synthesised and evaluated a monolithic and hydrophilic stir bar coating based on a methacrylic acid and divinylbenzene copolymer [poly(MAA-co-DVB)] for the SBSE of polar pharmaceuticals from complex environmental water samples. The extraction performance of the synthesised stir bar was compared to the extraction performance of a commercially available polydimethylsiloxane stir bar. The former produced significantly higher extraction efficiencies for polar analytes (% recovery values near 100% for most of the studied analytes) than the commercial product. The LODs of the developed method were 10 ng·L^−1^ for most of the target compounds, with the exception of naproxen (50 ng·L^−1^).

Similarly, the same authors [95] prepared a stir bar coated with a hydrophilic polymer based on poly(N-vinylpyrrolidone-co-divinylbenzene) for the sorptive extraction of polar pharmaceuticals from environmental water matrices, including river, effluent and influent waste water, followed by liquid desorption and subsequent LC-MS/MS. The LODs were between 10 and 50 ng·L^−1^.

Finally, a new polar monolith based on poly(poly(ethylene glycol) methacrylate-co-pentaerythritol triacrylate) (poly(PEGMA-co-PETRA)) was synthesised by Gilart *et al.* [96] and applied as a coating for stir bar sorptive extraction (SBSE) to determine a group of pharmaceuticals from environmental water samples. The coating could extract and desorb most of the studied analytes more effectively and rapidly than the recently commercialised polar stir bars. The analytical methodology was validated with LODs between 15 and 50 ng·L^−1^.

Martin *et al.* [97] compared SBSE and DLLME methods; both techniques were used with acetone as the dispersant and chlorobenzene as the extractant solvent to analyse statin drugs in different environmental water samples. DLLME generated better results than SBSE because SBSE only extracted two of the six pharmaceuticals. The DLLME recoveries approached 92%.

Different researchers have used DLLME to determine pharmaceutical compounds with LC-MS. Parrilla *et al.* [98] developed an ultrasound-assisted ionic liquid dispersive liquid-liquid microextraction (US-IL-DLLME) procedure for the extraction of nine pharmaceuticals from wastewater samples. The US process accelerated the formation of a fine cloudy solution containing an ionic liquid (IL), specifically 1-octyl-3-methylimidazolium hexafluorophosphate ([C_8_MIM][PF_6_]), and acetonitrile (ACN) as the extractant and dispersant, respectively. Moreover, the recoveries of the pharmaceuticals increased when an ice-water bath extraction was included during the analytical procedure. The LODs for the extraction of the target analytes from wastewater samples ranged from 0.2 to 60 ng·L^−1^ with recoveries between 88% and 111%.

**Table 1:** Microextraction techniques to determine pesticides in environmental water samples by liquid chromatography-tandem mass spectrometry.

Compounds	Matrix	Extraction Technique	Optimal Times	Handling	Recovery Accuracy (%)	LOD (ng·L^−1^)	Ref.
Organic tin compounds (trimethyltin chloride, tripropyltin chloride, tri-phenyltin hydroxide, tributyltin chloride)	Freshwater and seawater	SPME	Extraction: 45 min Desorption: 5 min	Easy to use	71–104	6–185	[59]
Benzylic and aliphatic quaternary ammonium compounds	Tap water and surface water	SPME	Extraction: 45 min Desorption: 15 min	96 well system	97–143	10–500	[60]
Polar pesticides (diuron, fluometuron, linuron, monuron, neburon, siduron, barban, carbaryl, chlorpropham, methiocarb, promecarb, propham)	Tap water, surface water and well water.	IT-SPME	15 draw/eject cycle 12 min	Lower handling	77–104	10–1200	[61]
Multiresidue (atrazine, chlorfenvinphos, chlorpyriphos, di(2-ethylhexyl)phthalate, diuron, isoproturon, simazine, terbuthylazine, trifluralin)	Wastewater, superficial and coastal water	IT-SPME	18 min	Lower handling	8–166	25–2500	[62]
Pesticides (alachlor, buprofezin, chlorpyriphos, chlorfenvinphos, diuron, fenthion, hexythiazox, isoproturon, malathion, tolclofos methyl, prochlora, imazalil, abamectin, diazinon, atrazine, simazine)	Surface water	SBSE	Extraction: 60 min Desorption: 30 min	Practical	3–62	10–1000	[63]
Antimicrobial compounds (triclosan, triclocarban)	River water and wastewater	SBSE	Extraction: 180 min Desorption: 15 min	Practical	25–89	2.5–10	[64]
Pesticides (carbofuran, clomazone, tebuconazole)	Tap water	DLLME	Extraction: seconds	Fast. Ease of operation	62.7–120	20	[65]
Triclosan and 2,4-dichlorophenol	Tap water and surface water	DLLME-SFO	Extraction: 1 min	Easy extraction-solidification	83–119	2–20	[66]
Triazine herbicides (cyanazine, simazine, atrazine)	Wastewater, river water underground water and drainage water	IL-DLPME	Extraction: 30 min Centrifugation: 15 min	Simple	85.1–100	50–60	[67]
Triclosan and triclocarban	Wastewater and tap water	IL-DLPME	Extraction: short time Centrifugation: 10 min	Simple	70.0–103.5	40–580	[68]

**Table 2:** Microextraction techniques to determine UV filters, alkyphenols, bisphenol A and PFCs in environmental water samples by liquid chromatography-tandem mass spectrometry.

Compounds	Matrix	Extraction Technique	Optimal Times	Handling	Recovery Accuracy (%)	LOD (ng·L^−1^)	Ref.
UV filters (2,2-dihydroxy-4-methoxybenzophenone, benzophenone-3, octocrylene, and octyldimethyl- p-aminobenzoic acid)	River water and wastewater	SBSE	Extraction: 180 min Desorption: 15 min	Practical	25–89	5–10	[64]
Benzotriazole UV stabilizers (UV P, UV 329, UV 326, UV 328, UV 327, UV 571, UV 360)	Seawater and wastewater	SBSE	Extraction: 120 min Desorption: 20 min	Practical	68.4–92.2	18.4–55.1	[71]
Personal care products (benzotriazole, 2,4-dihydroxybenzophenona, benzylparaben, 2,4-dihydroxy-4-methoxybenzophenone, benzophenone-3)	Wastewater	SBSE	Extraction: 240 min Desorption: 15 min (60 min for PA)	Optimal times depend on coatings	<1–80	5.0–10.0	[72]
BPA, APs	Seawater	DLLME	Extraction: 5 min Centrifugation: 3 min	Without any dispersant agent simplifies the process	84–104	5–30 (LOQ)	[74]
APs	Wastewater	HF-LPME	Extraction: 30 min		97–109	100 (LOQ)	[75]
PFOS and PFOA	Surface water and wastewater	IT-SPME	25 min	Lower handling 40 samples/day	81.1–85.4	1.5–3.2	[80]
PFOS and PFOA	River water	SPME	Extraction: 60 min Desorption: 15 min		88–120	2.5–7.5	[81]
PFOS	Tap, river and well water	VALLME	Extraction: 2 min Centrifugation: 2 min	Not require the use of certain sample preparation apparatus	90.8–105.1	1.6	[82]

**Table 3:** Microextraction techniques to determine hormones and pharmaceuticals in environmental water samples by liquid chromatography-tandem mass spectrometry.

Compounds	Matrix	Extraction Technique	Optimal Times	Handling	Recovery Accuracy (%)	LOD (ng·L^−1^)	Ref.
Estrogens (estrone, 17β-estradiol, estriol, ethynil estradiol, diethylstilbestrol)	Wastewater, river water	IT-SPME	20 draw/eject cycle 30 min	Lower handling 48 samples/day	86.1–106.8	2.7–11.7	[85]
Sulfonamide antibiotics (sulfaguanidine, sulfacetamide, sulfadiazine, sulfathiazine, sulfapyridine, sulfamerazine, sulfamethazine, sulfamethoxazole, sulfadimethoxine, sulfasalazine)	Wastewater	SPME	Extraction: 20 min Desorption: 30 min	Easy to use	29–229	9000–55300	[88]
Antibiotics (sulfamethazine, sulfisoxazole, sulfamethoxazole, sulfadimethoxine, sulfapyridine, trimethoprim, roxithromycin, erythromycin, clarithromycin)	Wastewater	SPME	Extraction: 30 min Desorption: 10 min	Easy to use	–	2.8–410.0	[89]
Analgesic and anti-inflammatory, antidepressant, antibiotics, lipid regulator, β-blockers, diuretics, ansiolitics, antiepileptic, antipsychotic	Wastewater	dSPME	Extraction: 30 min Desorption: 10 min	Minimizes laborious and complicated sample preparation procedures	89.2–109.7	5.0–50.0 (LOQ)	[90]
Pharmaceuticals (carbamazepine)	Wastewater	TFME	-	96 well-plate	–	–	[91]
Fluoroquinolones (enoxacin, ofloxacin, ciprofloxacin, norfloxacin, lomefloxacin)	Surface water and wastewater	IT-SPME	20 draw/eject cycles 30 min	Lower handling 48 samples/day	81.8–98	7.0–29.0	[92]
Non-steroidal anti-inflammatory drugs (acetaminophen, ibuprofen, naproxen, fenoprofn, flurbiprofen, loxoprofen, ketoprofen, mefenamic acid, flufenamic acid, diclofenac, tolfenamic acid, oxaprozin, phenylbutazone, indomethacin, acemetacin)	Surface water and wastewater	IT-SPME	20 draw/eject cycles 30 min	Lower handling 48 samples/day	80.4–100.4	5.0–65.0	[93]
Pharmaceuticals (paracetamol, naproxen, diclofenac, caffeine, antipyrine, propanolol, carbamazepine)	River water and wastewater	SBSE	Extraction: 240 min Desorption: 20 min	Practical	10–92	10.0–50.0	[94]
Pharmaceuticals (paracetamol, caffeine, antipyrine, propranolol, carbamazepine, ibuprofen, diclofenac)	River water and wastewater	SBSE	Extraction: 240 min Desorption: 15 min	Practical	9–110	10–50	[95]
Pharmaceuticals (paracetamol, caffeine, antipyrine, propranolol hydrochloride, pridinol methanesulfonate, carbamazepine, diclofenac)	Wastewater	SBSE	Extraction: 60 min Desorption 10 min	Better than commercial coatings	1–50	15–50	[96]
Statin drugs (atorvastatin, fluvastatin, lovastatin, pravastatin, rosuvastatin, simvastatin)	Pure water, wastewater and river water	DLLME	Centrifugation: 10 min (two times)	Faster	13–92	0.09–17.0	[97]
		SBSE	Extraction: 72 min Desorption: -		0–38	0.08	
Anti-inflammatory (paracetamol, ketoprofen, naproxen, ibuprofen, flufenamic acid, tolfenamic acid) β-blockers (metoprolol, bisoprolol, betaxolol)	Wastewater	US-IL-DLLME	Vortexed: 1 min Sonicated: 4 min Ice-water: 3 min Centrifugation: 8 min	Friendly	88–111	0.2–60.0	[98]
Antiinflammatory (diclofenac, ketoprofen, ibuprofen, naproxen)	River and tap water	DLLME	Sonicated: 1 min Centrifugation: 10 min (two times)	Simple and rapid	71–85	0.1–3.0	[99]
Clotrimazole	River water and wastewater	DLLME	Extraction: 1 min Centrifugation: 10 min		67.9–99.2	0.20–0.21	[100]
Acidic drugs (peroxicam, ketorolac, clofibric acid, naproxen, bezafibrate, fenoprofen, ibuprofen, diclofenac, indomethacin)	Wastewater	HF-LPME	Extraction: 45 min	Poor precision-manual operation	80–111	0.15–12.6	[101]
Antidepressant (amitriptyline, clomipramine, doxepin, mianserine, nortriptyline)	Wastewater	HF-LPME	Extraction: 120 min	Relatively simple	33–49	0.005–0.030	[102]
Antibiotic residue (erythromycin, spiramycin, tilmicosin, sulfathiazole, sulfamethazine, sulfamerazine, oxytetracycline, tetracycline, ciprofloxacin, danofloxacin, enrofloxacin)	River water	HF-LPME	Extraction: 60 min	Simple	79.2–118	10.0–250.0	[103]

Zgoła-Grześkowiak [99] used DLLME with LC-MS detection to extract anti-inflammatory pharmaceuticals from environmental samples. Chloroform was the extractant, and acetone was the dispersant. Under the optimised conditions, a two-step extraction with sonication was used; the LOQs ranged from 0.5 to 10 ng·L^−1^. Zgoła-Grześkowiak and Grześkowiak [100] developed a similar microextraction technique using ethanol as the dispersant and trichloroethylene as the extractant for the determination of clotrimazole in river water and wastewater effluent samples from wastewater treatment plants. The LOQ was approximately 0.7 ng·L^−1^.

Hollow fibre-protected liquid-phase microextraction (HF-LPME) was used by different researchers. Quintana *et al.* [101] used a hollow fibre liquid-phase microextraction (Accurel Q3/2 polypropylene tubular membranes) to extract/enrich acidic drugs from wastewater samples. After optimising the LPME method, very clean extracts could be obtained, avoiding signal suppression during the LC-MS/MS analysis of the analytes; the limits of quantification ranged from 0.5 to 42 ng·L^−1^. Additionally, Ho *et al.* [102] developed a similar technique able to accommodate large-sample-volume extractions in a single step for extracting antidepressant drugs from environmental waters. Compared to studies with small sample volumes, the closure of the hollow fibre and the type of liquid membrane were critical for large-volume extractions. Finally, Yudthavorasit *et al.* [103] used HF-LPME with UHPLC-MS/MS to determine 11 antibiotics in river water samples. The parameters were optimised to provide LODs from 10 to 250 ng·L^−1^. Good recoveries (79.2%–118%) were obtained using this technique, except in the study conducted by Ho *et al.* [102]. However, the authors obtained better enrichment factors by using large sample volumes, obtaining LODs in the range of pg·L^−1^.

## 5. Conclusions and Future Trends

LC–MS techniques are established methods for analysing organic micropollutants in environmental samples. These techniques can be applied to thermally labile compounds, and derivatisation is unnecessary for highly polar compounds. Mass analyser hybrid instruments that can identify metabolites and transformation products from their parent compounds have been introduced. Extraction techniques have also improved, and greener methodologies that consume less solvent have been introduced. Therefore, microextraction techniques can be combined with new extractants. Athough many ILs are not biodegradable and some are used as pesticides, they are safe extractants by low vapor pressure, and the improvements derived from these new materials, including MIPs and/or nanomaterials, and the development of novel devices are the most studied topics in analytical chemistry today. These advances might be economically and environmentally favourable because they decrease the environmental and economic impact of analytical chemistry laboratories, prevent exposure of the laboratory personnel to the vapours of harmful compounds and mitigate the problems caused by long and intensive sample pretreatments, which result in analyte losses and contamination [47].

## Acronyms

ACN: Acetonitrile
AMMWCNT-PDMS: Amino-modified multi-walled carbon nanotube-PDMS
APEOs: Alkylphenols ethoxylated
APs: Alkylphenols
BPA: Bisphenol A
BUVSs: Benzotriazole UV stabilizers
CCL: Contaminant candidate list
CME: Capillary microextraction
CNPrTEOS: Cyanopropyltriethoxysilane
CNTS: Carbon nanotubes
CW/DVB: Carbowax/divinylbenzene
CW/TPR: Carbowax/template resin
DAD: Diode array detector
DESI-MS: Desorption electrospray ionization mass spectrometry
DI-SPME: Direct inmersion solid phase microextraction
DLLME: Dispersive liquid-liquid microextraction
DLLME-SFO: DLLME based on floating organic droplet
DLPME: Dispersive liquid phase microextraction
DSDME: Directly-suspended droplet microextraction
dSPME: dual-SPME
EDCs: Endocrine disruptor compounds
EG: Ethyleneglycol
ESI: Electrospray ionization
EU: European Union
FD: Fluorescence detector
FDA: Food and Drug Administration
FQs: Fluoroquinolones
GC: Gas chromatography
HF(2)ME: Hollow-fibre-protected 2-phase microextraction
HF(3)ME: Hollow-fibre-protected 3-phase microextraction
HF-LPME: Hollow-fibre liquid phase microextraction
HFM-LLLME: Hollow membrane liquid-liquid-liquid microextraction
HF-SLPME: Hollow fibre solid-liquid phase microextraction
HPLC: High performance liquid chromatography
HS-SDME: Headspace single-drop microextraction
HS-SPME: Headspace solid phase microextraction
ICP-MS: Inductively coupled plasma-mass spectrometry
IL-DLLME: Ionic liquid-dispersive liquid-liquid microextraction
IL-DLPME: Ionic liquid dispersive liquid-phase microextraction
ILs: Ionic liquids
IT-SPME: In-tube solid phase microextraction
LC-MS/MS: Liquid chromatography tandem mass spectrometry
LC-MS: Liquid chromatography-mass spectrometry
LLE: Liquid-liquid extraction
LLLME: Liquid-liquid-liquid microextraction
LLME: Liquid-liquid microextraction
LODs: Limit of detections
LOQs: Limit of quantifications
LPME: Liquid-phase microextraction
MeOH: Methanol
MIPs: Moleculary-imprinted polymers
MISPME: Moleculary-imprinted solid phase microextraction
MOF: Metal-organic framework
MS/MS: Tandem MS
MS: Mass spectrometry
MWCNTs: Multi-wall carbon nanotubes
NSAIDs: Non-steroidal anti-inflammatory drugs
PAHs: Polyciclic aromatic hydrocarbons
PCBs: Polychlorinated biphenyls
PCPs: Personal care products
PDMS/DVB: Polydimethylsiloxane/divinibenzene
PDMS: Polydimethylsiloxane
PEG: Polyethyleneglycol
PFCs: Perfluorinated compounds
PFOA: Perfluorooctanoic acid
PFOS: Perfluorooctane sulfonate
PILs: Polymeric ionic liquids
POP: Persistent organic pollutants
PPCPs: Pharmaceuticals and personal care products
PPY: Polypyrrole
RDSE: Rotating disk sorptive extraction
SBSE: Stir-bar sorptive extraction
SDCME: Single-drop coacervative microextraction
SDME: Single-drop microextraction
SME: Solven microextraction
SPE: Solid phase extraction
SPME: Solid phase microextraction
SWCNTs: Single-wall carbon nanotubes
TFME: Thin-film microextraction
TF-SPME: Thin-film solid phase microextraction
TOF/MS: Time-of-flight mass spectrometry
UHPLC: Ultra high performance liquid chromatography
UHPLC-MS/MS: Ultra high performance liquid chromatography tandem mass spectrometry
UHPLC-MS: Ultra high performance liquid chromatography mass spectrometry
USEPA: US Environmental Protection Agency
US-IL-DLLME: Ultrasound-assisted ionic liquid dispersive liquid-liquid microextraction
VALLME: Vortex-Assisted liquid–liquid Microextraction
WFD: Water Framework Directive
WWTP: Wastewater treatment plant
